# 1642. Vancomycin AUC_24_ Estimation Using First Order Pharmacokinetic Methods in Pediatric Patients

**DOI:** 10.1093/ofid/ofad500.1476

**Published:** 2023-11-27

**Authors:** Katie L Wallace, David Burgess, Katie B Olney, Hope Brandon

**Affiliations:** University of Kentucky HealthCare, Lexington, KY; UK HealthCare, Lexington, KY; University of Kentucky HealthCare, Lexington, KY; University of Kentucky HealthCare, Lexington, KY

## Abstract

**Background:**

The 2020 guidelines for the monitoring of vancomycin emphasize the importance of timely assessment of AUC_24_ for both efficacy and safety in pediatric patients. However, real-world data supporting the feasibility of vancomycin AUC_24_ in pediatric patients using first-order equations is lacking.

**Figure:**
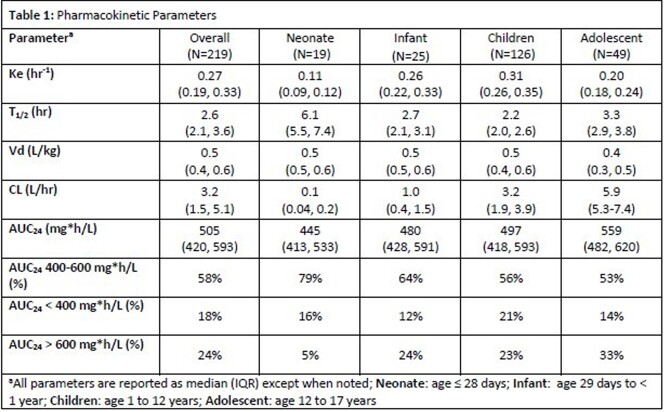

**Methods:**

This is a single-center, retrospective cohort study of hospitalized pediatric patients (< 18 years) receiving intravenous (IV) vancomycin between 1/1/2020 and 8/20/2022. Patients were included if they received at least 24 hours of IV vancomycin with peak and trough concentrations obtained in the first 96 hours of therapy. Patients with baseline renal dysfunction were excluded. First-order equations were utilized to estimate K_e_, V_d_, Cl, and AUC_24._

**Results:**

Overall, 219 patients (68% male, 87% Caucasian, 24% critically ill, median age of 6 years (IQR 1-12)) met inclusion criteria. Of the total patients, 9% were neonates (age ≤ 28 days), 11% were infants (age 29 days to < 1 year), 58% were children (age 1 to 12 years), and 22% were adolescents (age 13 to 17 years). The pharmacokinetic parameters for each group are outlined in **Table 1**. The estimated AUC_24_ was within the therapeutic range of 400-600 mg*hr/L for the majority of patients. However, when comparing age groups, adolescents had the lowest (53%) while neonates had the greatest (79%) percentage of patients within the therapeutic range

**Conclusion:**

Our results reinforce the variability in vancomycin PK seen across pediatric age groups, confirming the need to tailor empiric dosing regimens by patient age. For patients with AUC_24_ outside the therapeutic range, more patients had SUPRAtherapeutic rather than SUBtherapeutic AUC_24_. In response to results of this study, institutional empiric vancomycin dosing will be decreased for all age groups outside the neonatal group to better align with the 2020 guideline recommended AUC_24_ of 400 - 600 mg*hr/L for pediatric patients.

**Disclosures:**

**Katie B. Olney, PharmD, BCIDP**, The Society of Infectious Diseases Pharmacists (SIDP): Grant/Research Support

